# *N*-(4-Eth­oxy­phen­yl)-3-oxobutanamide

**DOI:** 10.1107/S2414314623005655

**Published:** 2023-07-04

**Authors:** Sreevandana Yerramsetty, Frank R. Fronczek, Rao M. Uppu

**Affiliations:** a Department of Environmental Toxicology, Southern University and A&M College, Baton Rouge, LA 70813, USA; b Department of Chemistry, Louisiana State University, Baton Rouge, LA 70803, USA; University of Aberdeen, United Kingdom

**Keywords:** crystal structure, metabolic inter­mediates, nonopiod analgesics, phenacetin congeners

## Abstract

The title compound, C_12_H_15_NO_3_, crystallizes with *Z*′ = 2 in space group *Pca*2_1_ with the two independent mol­ecules having almost the same conformation, differing mostly at the end of the butanamide chain. A local inversion center near 1/8, 3/4, *z* relates the two mol­ecules, as is common for structures in this space group with *Z*′ = 2. The mol­ecule crystallizes as the keto tautomer, and the β-diketone moieties are twisted out of planarity, with O—C⋯C—O pseudo torsion angles of −74.4 (5) and −83.9 (5)°. The N—H group of each independent mol­ecule donates an inter­molecular hydrogen bond to an amide carbonyl oxygen atom by positive or negative translations along the *b* axis, thus forming anti­parallel chains propagating in the [010] direction.

## Abstract 2

The title compound, C_12_H_15_NO_3_, crystallizes with *Z*′ = 2 in space group *Pca*2_1_ with the two independent mol­ecules having almost the same conformation, differing mostly at the end of the butanamide chain. A local inversion center near 1/8, 3/4, *z* relates the two mol­ecules, as is common for structures in this space group with *Z*′ = 2. The mol­ecule crystallizes as the keto tautomer, and the β-diketone moieties are twisted out of planarity, with O—C⋯C—O pseudo torsion angles of −74.4 (5) and −83.9 (5)°.

## Graphical abstract

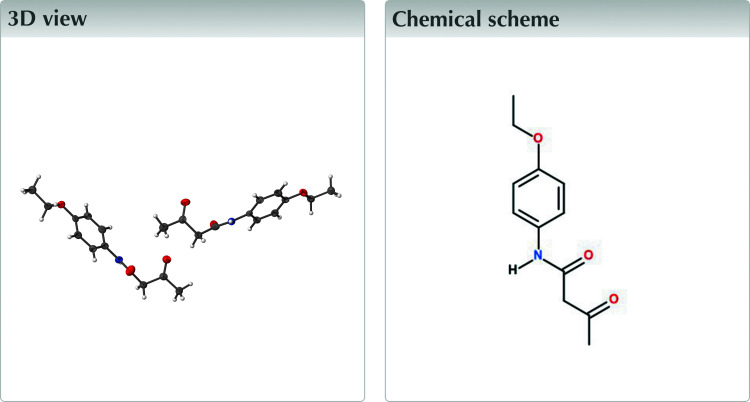

## Structure description

*N*-(4-Eth­oxy­phen­yl)-3-oxobutanamide is a putative inter­mediate in the biotransformation of bucetin [*N*-(4-eth­oxy­phen­yl)-3-hy­droxy­butanamide], an analgesic–anti­pyretic once considered to be a safer alternative for phenacetin (Fujimura & Shinozaki, 1996 ▸; Grüssner & Schnider, 1996 ▸; Togei *et al.*, 1987 ▸). Shibasaki *et al.* (1968 ▸) demonstrated that approximately 62% of orally administered bucetin in rabbits is converted to glucuronides of *N*-(4-hy­droxy­phen­yl)-3-oxobutanamide, *N*-(4-hy­droxy­phen­yl)-3-hy­droxy­butanamide, and *N*-(4-hy­droxy­phen­yl)acetamide. Intra­venous administration of bucetin, on the other hand, mainly resulted in the formation of the glucuronide of *N*-(4-hy­droxy­phen­yl)acetamide, with a maximum yield of 98%. These findings indicate that oxidative *O*-de-­ethyl­ation, keto conversion, and γ-deca­rboxylation are involved in the biotransformation of bucetin, leading to the endogenous production of *N*-(4-hy­droxy­phen­yl)acetamide, the relevant analgesic compound. However, the specific order of *O*-de-ethyl­ation and keto conversion remains uncertain (Shibasaki *et al.*, 1968 ▸).

The mol­ecular structure of *N*-(4-eth­oxy­phen­yl)-3-oxobutanamide, C_12_H_15_NO_3_, contains a β-diketone functionality that is similar in nature to the one present in linear and cyclic 1,3-diketone compounds (Hansen, 2021 ▸; Shokova *et al.*, 2015 ▸). Understandably, the diketone functionality also exists in its enol tautomeric form. This structural characteristic makes the amide side chain susceptible to electrophilic substitution reactions, particularly with oxidizing agents in the cellular milieu such as per­oxy­nitrite (*O*=NOO^−^)-per­oxy­nitrous acid (O=NOOH; p*K*_a_ ≃ 6.8) and hypochlorite (^−^OCl)-hypo­chlorous acid (HOCl; p*K*_a_ ≃ 7.5) conjugate acid–base systems (Agu *et al.*, 2020 ▸; Uppu & Pryor, 1996 ▸; Zhang & Banwell, 2011 ▸). Furthermore, the keto conversion process of bucetin eliminates the chiral center, potentially facilitating the formation of various types of metal-ion chelates (Basak & Singh, 2015 ▸; Karki *et al.*, 2016 ▸). To further comprehend the processes and potential implications for the overall toxicity of bucetin and its congeners, in the present study, the crystal structure of the title compound is reported.

**Figure 1:**
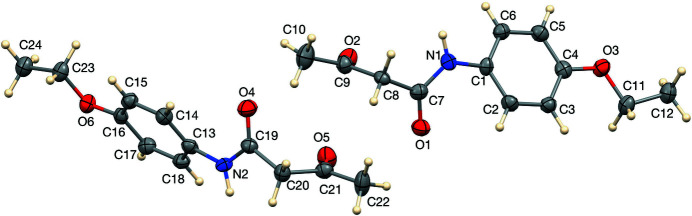
The asymmetric unit of the title compound showing 50% displacement ellipsoids.

**Figure 2:**
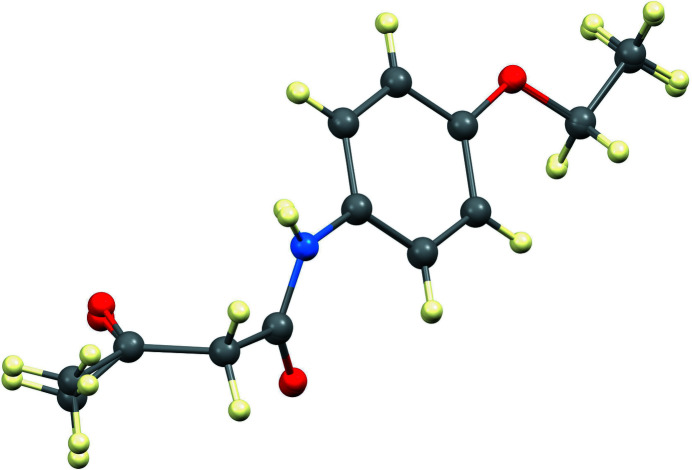
Overlay of the two independent mol­ecules.

The title compound, shown in Fig. 1 ▸, crystallizes with two independent mol­ecules in the asymmetric unit. The conformations of the two mol­ecules are quite similar, with the largest difference being at the end of the butanamide chain (O2—C9—C10 and O5—C21—C22). An overlay of the two mol­ecules (Fig. 2 ▸) shows the small difference, with r.m.s. deviation = 0.10 Å and maximum deviation 0.30 (1) Å for C10⋯C22. This small difference in conformation can also be seen in torsion angles describing the twist of the β-diketone units, −74.4 (5)° for O1—C7⋯C9—O2 and −83.9 (5)° for O4—C19⋯C21—O5.

**Figure 3:**
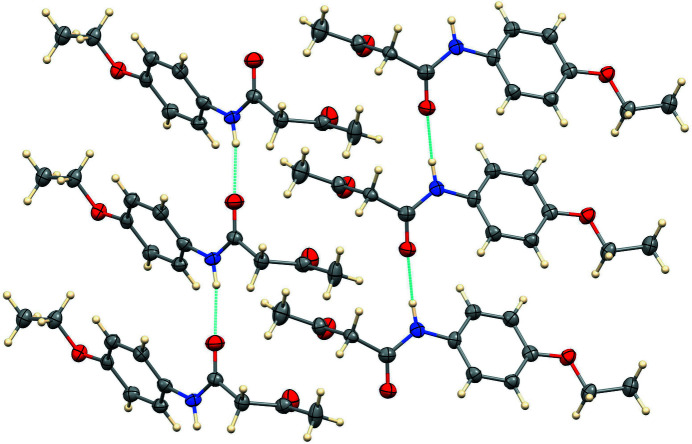
Partial packing diagram with N—H⋯O hydrogen bonds shown as blue lines.

The N—H moiety in both mol­ecules donates an inter­molecular hydrogen bond (Table 1 ▸) to amide carbonyl oxygen atoms as shown in Fig. 3 ▸. The N1⋯O1 (at *x*, *y* – 1, *z*) distance is 2.878 (6) Å and the N2⋯O4 (at *x*, *y* + 1, *z*) distance is 2.856 (6) Å. Thus, the two independent mol­ecules form anti­parallel chains in the [010] direction, as shown in Fig. 3 ▸.

**Figure 4:**
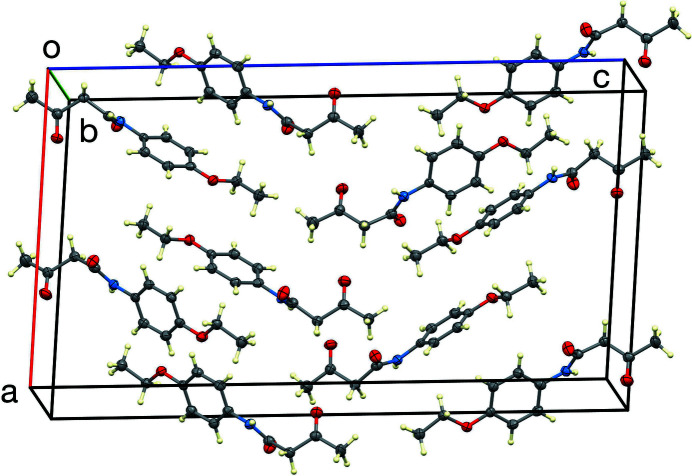
The unit-cell packing, viewed approximately down [010].

The unit cell is illustrated in Fig. 4 ▸, which shows local approximate inversion centers at 0.123 0.737, 0.750 and 0.623, 0.263, 0.750. Marsh *et al.* (1998 ▸) have shown that approximately 75% of structures with *Z*′ = 2 in space groups *Pca*2_1_ and *Pna*2_1_ have such local centers and that in *Pca*2_1_, the local centers tend to be near 1/8, 1/4, *z*. This agrees well with what we observe in the title structure, after an origin shift of *x* – 1/2 or *y* – 1/2.

## Synthesis and crystallization

*N*-(4-Eth­oxy­phen­yl)-3-oxobutanamide, C_12_H_15_NO_3 (_CAS No. 122–87-2) was obtained from AmBeed, Arlington Heights, IL, USA and was used without further purification. Crystals in the form of colorless laths were prepared by slow cooling of a nearly saturated solution of the title compound in boiling deionized water.

## Refinement

Crystal data, data collection and structure refinement details are summarized in Table 2 ▸.

## Supplementary Material

Crystal structure: contains datablock(s) I. DOI: 10.1107/S2414314623005655/hb4435sup1.cif

Structure factors: contains datablock(s) I. DOI: 10.1107/S2414314623005655/hb4435Isup2.hkl

Supporting information file. DOI: 10.1107/S2414314623005655/hb4435Isup3.cml

CCDC reference: 2276880

Additional supporting information:  crystallographic information; 3D view; checkCIF report

## Figures and Tables

**Table 1:** Hydrogen-bond geometry (Å, °)

*D*—H⋯*A*	*D*—H	H⋯*A*	*D*⋯*A*	*D*—H⋯*A*
N1—H1*N*⋯O1^i^	0.92 (6)	1.98 (6)	2.878 (6)	165 (5)
N2—H2*N*⋯O4^ii^	0.89 (7)	1.98 (7)	2.856 (6)	168 (5)

Symmetry codes: (i) 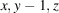 ; (ii) 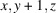 .

**Table 2:** Experimental details

Crystal data	
Chemical formula	C_12_H_15_NO_3_
*M*_r_	221.25
Crystal system, space group	Orthorhombic, *P* *c* *a*2_1_
Temperature (K)	100
*a*, *b*, *c* (Å)	16.4113 (8), 4.9076 (3), 28.8889 (15)
*V* (Å^3^)	2326.7 (2)
*Z*	8
Radiation type	Cu *K*α
μ (mm^−1^)	0.75
Crystal size (mm)	0.31 × 0.09 × 0.03

Data collection	
Diffractometer	Bruker Kappa APEXII CCD DUO
Absorption correction	Multi-scan (*SADABS*; Krause *et al.*, 2015 ▸)
*T*_min_, *T*_max_	0.732, 0.978
No. of measured, independent and observed [*I* > 2σ(*I*)] reflections	16274, 4191, 3503
*R*_int_	0.061
(sin θ/λ)_max_ (Å^−1^)	0.603

Refinement	
*R*[*F* ^2^ > 2σ(*F* ^2^)], *wR*(*F* ^2^), *S*	0.058, 0.156, 1.04
No. of reflections	4191
No. of parameters	299
No. of restraints	1
H-atom treatment	H atoms treated by a mixture of independent and constrained refinement
Δρ_max_, Δρ_min_ (e Å^−3^)	0.20, −0.32
Absolute structure	Flack *x* determined using 1426 quotients [(*I* ^+^)−(*I* ^−^)]/[(*I* ^+^)+(*I* ^−^)] (Parsons *et al.*, 2013 ▸).
Absolute structure parameter	0.3 (3)

Computer programs: *APEX2* and *SAINT* (Bruker, 2016 ▸), *SHELXT2014/5* (Sheldrick, 2008 ▸), *SHELXL2017/1* (Sheldrick, 2015 ▸), *Mercury* (Macrae *et al.*, 2020 ▸), and *publCIF* (Westrip, 2010 ▸).

## full crystallographic data

### Crystal data

**Table:**

C_12_H_15_NO_3_	*D*_x_ = 1.263 Mg m^−^^3^
*M**_r_* = 221.25	Cu *K*α radiation, λ = 1.54184 Å
Orthorhombic, *P**c**a*2_1_	Cell parameters from 3285 reflections
*a* = 16.4113 (8) Å	θ = 3.1–68.1°
*b* = 4.9076 (3) Å	µ = 0.75 mm^−^^1^
*c* = 28.8889 (15) Å	*T* = 100 K
*V* = 2326.7 (2) Å^3^	Lath, colourless
*Z* = 8	0.31 × 0.09 × 0.03 mm
*F*(000) = 944	

### Data collection

**Table:**

Bruker Kappa APEXII CCD DUO diffractometer	4191 independent reflections
Radiation source: IµS microfocus	3503 reflections with *I* > 2σ(*I*)
QUAZAR multilayer optics monochromator	*R*_int_ = 0.061
φ and ω scans	θ_max_ = 68.4°, θ_min_ = 3.1°
Absorption correction: multi-scan (SADABS; Krause *et al.*, 2015)	*h* = −19→19
*T*_min_ = 0.732, *T*_max_ = 0.978	*k* = −5→5
16274 measured reflections	*l* = −34→34

### Refinement

**Table:**

Refinement on *F*^2^	Hydrogen site location: mixed
Least-squares matrix: full	H atoms treated by a mixture of independent and constrained refinement
*R*[*F*^2^ > 2σ(*F*^2^)] = 0.058	*w* = 1/[σ^2^(*F*_o_^2^) + (0.1042*P*)^2^] where *P* = (*F*_o_^2^ + 2*F*_c_^2^)/3
*wR*(*F*^2^) = 0.156	(Δ/σ)_max_ < 0.001
*S* = 1.04	Δρ_max_ = 0.20 e Å^−^^3^
4191 reflections	Δρ_min_ = −0.32 e Å^−^^3^
299 parameters	Absolute structure: Flack *x* determined using 1426 quotients [(*I*^+^)-(*I*^-^)]/[(*I*^+^)+(*I*^-^)] (Parsons *et al.*, 2013).
1 restraint	Absolute structure parameter: 0.3 (3)

### Special details

**Table:**

Geometry. All esds (except the esd in the dihedral angle between two l.s. planes) are estimated using the full covariance matrix. The cell esds are taken into account individually in the estimation of esds in distances, angles and torsion angles; correlations between esds in cell parameters are only used when they are defined by crystal symmetry. An approximate (isotropic) treatment of cell esds is used for estimating esds involving l.s. planes.
Refinement. All H atoms were located in difference maps and those on C were thereafter treated as riding in geometrically idealized positions with C—H distances of 0.95 Å for phenyl, 0.99 Å for CH_2_, and 0.98 Å for methyl. The coordinates of the N-bound H atoms were refined. *U*_iso_(H) values were assigned as 1.2*U*_eq_ for the attached atom (1.5 for methyl).

### Fractional atomic coordinates and isotropic or equivalent isotropic displacement parameters (Å^2^)

**Table:**

	*x*	*y*	*z*	*U*_iso_*/*U*_eq_	
O1	0.4059 (2)	0.8722 (7)	0.59122 (13)	0.0370 (8)	
O2	0.3364 (2)	0.5583 (9)	0.49998 (18)	0.0416 (10)	
O3	0.1960 (2)	0.5495 (8)	0.77556 (15)	0.0318 (9)	
N1	0.3749 (2)	0.4356 (10)	0.61261 (17)	0.0279 (9)	
H1N	0.376 (3)	0.257 (13)	0.603 (2)	0.034*	
C1	0.3280 (4)	0.4895 (9)	0.6532 (2)	0.0253 (13)	
C2	0.3476 (3)	0.6947 (10)	0.68408 (16)	0.0291 (10)	
H2	0.390918	0.816564	0.677246	0.035*	
C3	0.3040 (3)	0.7231 (10)	0.72522 (17)	0.0284 (9)	
H3	0.317128	0.865235	0.746269	0.034*	
C4	0.2411 (3)	0.5422 (10)	0.7353 (2)	0.0271 (12)	
C5	0.2219 (3)	0.3388 (10)	0.70439 (17)	0.0309 (10)	
H5	0.178674	0.216337	0.711170	0.037*	
C6	0.2650 (3)	0.3111 (10)	0.66356 (16)	0.0286 (9)	
H6	0.251432	0.169414	0.642516	0.034*	
C7	0.4104 (2)	0.6254 (10)	0.58497 (18)	0.0295 (10)	
C8	0.4576 (4)	0.5086 (9)	0.5444 (3)	0.0255 (12)	
H8A	0.507672	0.617418	0.539584	0.031*	
H8B	0.474213	0.319620	0.551754	0.031*	
C9	0.4082 (4)	0.5078 (10)	0.5000 (3)	0.0323 (13)	
C10	0.4544 (4)	0.4355 (16)	0.4575 (3)	0.0487 (15)	
H10A	0.416301	0.407453	0.431853	0.073*	
H10B	0.485339	0.267644	0.462942	0.073*	
H10C	0.492058	0.583596	0.449776	0.073*	
C11	0.2132 (3)	0.7601 (11)	0.80814 (18)	0.0326 (10)	
H11A	0.198941	0.940344	0.795033	0.039*	
H11B	0.271821	0.760281	0.816206	0.039*	
C12	0.1621 (3)	0.7016 (11)	0.85056 (17)	0.0370 (11)	
H12A	0.104273	0.701628	0.842045	0.055*	
H12B	0.171948	0.842421	0.873961	0.055*	
H12C	0.176886	0.523000	0.863171	0.055*	
O4	0.6561 (2)	0.6038 (8)	0.40666 (14)	0.0420 (8)	
O5	0.5875 (2)	0.9503 (9)	0.49781 (19)	0.0435 (10)	
O6	0.4501 (2)	0.9546 (7)	0.22415 (15)	0.0301 (8)	
N2	0.6290 (3)	1.0406 (9)	0.38715 (18)	0.0264 (10)	
H2N	0.641 (3)	1.208 (13)	0.397 (2)	0.032*	
C13	0.5832 (4)	0.9997 (9)	0.3459 (3)	0.0276 (14)	
C14	0.6010 (3)	0.7971 (10)	0.31464 (16)	0.0288 (10)	
H14	0.643442	0.671498	0.321243	0.035*	
C15	0.5576 (3)	0.7736 (10)	0.27343 (16)	0.0297 (10)	
H15	0.570438	0.632640	0.252109	0.036*	
C16	0.4955 (3)	0.9561 (10)	0.2636 (2)	0.0261 (11)	
C17	0.4762 (3)	1.1597 (11)	0.29542 (17)	0.0294 (10)	
H17	0.433225	1.283796	0.289051	0.035*	
C18	0.5197 (3)	1.1807 (10)	0.33630 (16)	0.0291 (10)	
H18	0.506319	1.319142	0.357984	0.035*	
C19	0.6629 (3)	0.8490 (10)	0.41366 (16)	0.0276 (9)	
C20	0.7110 (4)	0.9482 (12)	0.4546 (2)	0.0309 (12)	
H20A	0.731238	1.134403	0.448221	0.037*	
H20B	0.758851	0.828301	0.459146	0.037*	
C21	0.6607 (3)	0.9520 (11)	0.4987 (3)	0.0314 (12)	
C22	0.7074 (4)	0.9522 (15)	0.5434 (3)	0.0468 (16)	
H22A	0.670234	0.993332	0.569083	0.070*	
H22B	0.750265	1.090863	0.542116	0.070*	
H22C	0.732011	0.772620	0.548316	0.070*	
C23	0.4662 (3)	0.7422 (10)	0.19102 (18)	0.0321 (10)	
H23A	0.451954	0.562321	0.204233	0.038*	
H23B	0.524691	0.740870	0.182612	0.038*	
C24	0.4147 (3)	0.8001 (11)	0.14883 (17)	0.0360 (11)	
H24A	0.357062	0.805463	0.157740	0.054*	
H24B	0.423055	0.656115	0.125810	0.054*	
H24C	0.430410	0.976165	0.135568	0.054*	

### Atomic displacement parameters (Å^2^)

**Table:**

	*U*^11^	*U*^22^	*U*^33^	*U*^12^	*U*^13^	*U*^23^
O1	0.0448 (18)	0.028 (2)	0.0384 (19)	−0.0009 (15)	0.0072 (15)	−0.0003 (16)
O2	0.0267 (19)	0.059 (2)	0.039 (2)	0.0075 (17)	−0.0055 (16)	−0.007 (2)
O3	0.0274 (18)	0.0360 (19)	0.032 (2)	−0.0007 (14)	0.0038 (15)	−0.0065 (17)
N1	0.027 (2)	0.029 (2)	0.028 (2)	0.0025 (16)	−0.0001 (18)	0.003 (2)
C1	0.020 (2)	0.034 (3)	0.022 (3)	0.0030 (16)	−0.003 (2)	0.0009 (16)
C2	0.0237 (19)	0.032 (3)	0.031 (2)	−0.0030 (17)	−0.0004 (17)	0.0022 (19)
C3	0.029 (2)	0.028 (2)	0.029 (2)	−0.0017 (17)	−0.0030 (18)	−0.0004 (19)
C4	0.021 (2)	0.029 (2)	0.031 (3)	0.0067 (17)	−0.001 (2)	0.004 (2)
C5	0.0256 (19)	0.032 (2)	0.035 (2)	−0.0019 (18)	0.0008 (17)	0.002 (2)
C6	0.027 (2)	0.028 (2)	0.030 (2)	0.0009 (18)	−0.0037 (18)	−0.0004 (18)
C7	0.026 (2)	0.029 (3)	0.034 (2)	0.0003 (18)	−0.0044 (18)	−0.001 (2)
C8	0.025 (2)	0.022 (2)	0.030 (3)	0.0018 (15)	0.001 (2)	0.0024 (16)
C9	0.032 (3)	0.036 (3)	0.029 (3)	0.0005 (17)	−0.002 (2)	0.0024 (18)
C10	0.038 (3)	0.073 (4)	0.036 (3)	0.008 (3)	−0.002 (3)	−0.004 (3)
C11	0.027 (2)	0.041 (3)	0.030 (2)	0.002 (2)	0.0008 (18)	−0.005 (2)
C12	0.036 (2)	0.045 (3)	0.029 (2)	0.002 (2)	0.002 (2)	−0.002 (2)
O4	0.057 (2)	0.030 (2)	0.0396 (19)	0.0004 (16)	−0.0100 (17)	−0.0013 (16)
O5	0.031 (2)	0.061 (3)	0.039 (2)	−0.0039 (16)	−0.0005 (17)	0.006 (2)
O6	0.0274 (17)	0.0349 (18)	0.0280 (19)	0.0050 (14)	−0.0025 (15)	−0.0017 (16)
N2	0.026 (2)	0.024 (2)	0.028 (2)	−0.0023 (15)	−0.0042 (17)	−0.0044 (17)
C13	0.024 (3)	0.027 (3)	0.032 (4)	−0.0048 (15)	−0.001 (2)	0.0032 (17)
C14	0.0260 (19)	0.030 (3)	0.030 (2)	0.0006 (18)	−0.0009 (18)	0.0012 (19)
C15	0.028 (2)	0.032 (3)	0.029 (2)	−0.0021 (18)	0.0019 (18)	−0.0038 (18)
C16	0.023 (2)	0.033 (2)	0.023 (3)	−0.0039 (18)	0.003 (2)	0.001 (2)
C17	0.026 (2)	0.029 (2)	0.034 (2)	0.0025 (18)	0.0022 (16)	−0.002 (2)
C18	0.030 (2)	0.024 (2)	0.033 (2)	−0.0001 (18)	0.0034 (18)	−0.0014 (19)
C19	0.027 (2)	0.026 (3)	0.031 (2)	0.0010 (18)	−0.0004 (17)	0.0006 (19)
C20	0.027 (2)	0.037 (3)	0.029 (3)	−0.001 (2)	0.001 (2)	0.000 (2)
C21	0.028 (3)	0.032 (3)	0.035 (3)	−0.0023 (19)	0.000 (2)	−0.001 (2)
C22	0.034 (3)	0.081 (4)	0.025 (3)	−0.005 (3)	−0.002 (2)	0.001 (3)
C23	0.026 (2)	0.041 (3)	0.029 (2)	0.0041 (19)	0.0003 (18)	−0.004 (2)
C24	0.033 (2)	0.044 (3)	0.030 (2)	0.001 (2)	−0.0009 (19)	−0.004 (2)

### Geometric parameters (Å, º)

**Table:**

O1—C7	1.227 (6)	O4—C19	1.226 (6)
O2—C9	1.205 (7)	O5—C21	1.201 (7)
O3—C4	1.379 (8)	O6—C16	1.361 (8)
O3—C11	1.426 (6)	O6—C23	1.440 (6)
N1—C7	1.358 (7)	N2—C19	1.334 (7)
N1—C1	1.427 (8)	N2—C13	1.424 (9)
N1—H1N	0.92 (6)	N2—H2N	0.89 (7)
C1—C2	1.383 (8)	C13—C14	1.375 (8)
C1—C6	1.388 (8)	C13—C18	1.396 (7)
C2—C3	1.394 (7)	C14—C15	1.392 (7)
C2—H2	0.9500	C14—H14	0.9500
C3—C4	1.393 (7)	C15—C16	1.386 (8)
C3—H3	0.9500	C15—H15	0.9500
C4—C5	1.376 (8)	C16—C17	1.394 (7)
C5—C6	1.382 (7)	C17—C18	1.384 (7)
C5—H5	0.9500	C17—H17	0.9500
C6—H6	0.9500	C18—H18	0.9500
C7—C8	1.518 (8)	C19—C20	1.503 (8)
C8—C9	1.516 (10)	C20—C21	1.518 (9)
C8—H8A	0.9900	C20—H20A	0.9900
C8—H8B	0.9900	C20—H20B	0.9900
C9—C10	1.485 (11)	C21—C22	1.502 (10)
C10—H10A	0.9800	C22—H22A	0.9800
C10—H10B	0.9800	C22—H22B	0.9800
C10—H10C	0.9800	C22—H22C	0.9800
C11—C12	1.513 (7)	C23—C24	1.511 (7)
C11—H11A	0.9900	C23—H23A	0.9900
C11—H11B	0.9900	C23—H23B	0.9900
C12—H12A	0.9800	C24—H24A	0.9800
C12—H12B	0.9800	C24—H24B	0.9800
C12—H12C	0.9800	C24—H24C	0.9800
			
C4—O3—C11	118.0 (4)	C16—O6—C23	117.4 (4)
C7—N1—C1	125.9 (5)	C19—N2—C13	127.0 (4)
C7—N1—H1N	118 (4)	C19—N2—H2N	112 (4)
C1—N1—H1N	116 (4)	C13—N2—H2N	121 (4)
C2—C1—C6	119.6 (6)	C14—C13—C18	119.2 (6)
C2—C1—N1	122.6 (5)	C14—C13—N2	122.6 (5)
C6—C1—N1	117.5 (5)	C18—C13—N2	118.1 (5)
C1—C2—C3	120.2 (5)	C13—C14—C15	120.9 (5)
C1—C2—H2	119.9	C13—C14—H14	119.6
C3—C2—H2	119.9	C15—C14—H14	119.6
C4—C3—C2	119.7 (5)	C16—C15—C14	119.9 (5)
C4—C3—H3	120.2	C16—C15—H15	120.1
C2—C3—H3	120.2	C14—C15—H15	120.1
C5—C4—O3	116.3 (5)	O6—C16—C15	124.8 (5)
C5—C4—C3	119.8 (5)	O6—C16—C17	115.6 (5)
O3—C4—C3	123.9 (5)	C15—C16—C17	119.6 (5)
C4—C5—C6	120.6 (5)	C18—C17—C16	119.9 (5)
C4—C5—H5	119.7	C18—C17—H17	120.0
C6—C5—H5	119.7	C16—C17—H17	120.0
C5—C6—C1	120.2 (5)	C17—C18—C13	120.4 (5)
C5—C6—H6	119.9	C17—C18—H18	119.8
C1—C6—H6	119.9	C13—C18—H18	119.8
O1—C7—N1	124.4 (5)	O4—C19—N2	124.0 (4)
O1—C7—C8	121.1 (4)	O4—C19—C20	119.7 (4)
N1—C7—C8	114.5 (4)	N2—C19—C20	116.3 (4)
C9—C8—C7	112.4 (5)	C19—C20—C21	112.3 (5)
C9—C8—H8A	109.1	C19—C20—H20A	109.1
C7—C8—H8A	109.1	C21—C20—H20A	109.1
C9—C8—H8B	109.1	C19—C20—H20B	109.1
C7—C8—H8B	109.1	C21—C20—H20B	109.1
H8A—C8—H8B	107.8	H20A—C20—H20B	107.9
O2—C9—C10	123.2 (7)	O5—C21—C22	121.9 (7)
O2—C9—C8	121.5 (7)	O5—C21—C20	121.7 (7)
C10—C9—C8	115.2 (5)	C22—C21—C20	116.4 (5)
C9—C10—H10A	109.5	C21—C22—H22A	109.5
C9—C10—H10B	109.5	C21—C22—H22B	109.5
H10A—C10—H10B	109.5	H22A—C22—H22B	109.5
C9—C10—H10C	109.5	C21—C22—H22C	109.5
H10A—C10—H10C	109.5	H22A—C22—H22C	109.5
H10B—C10—H10C	109.5	H22B—C22—H22C	109.5
O3—C11—C12	106.7 (4)	O6—C23—C24	107.3 (4)
O3—C11—H11A	110.4	O6—C23—H23A	110.3
C12—C11—H11A	110.4	C24—C23—H23A	110.3
O3—C11—H11B	110.4	O6—C23—H23B	110.3
C12—C11—H11B	110.4	C24—C23—H23B	110.3
H11A—C11—H11B	108.6	H23A—C23—H23B	108.5
C11—C12—H12A	109.5	C23—C24—H24A	109.5
C11—C12—H12B	109.5	C23—C24—H24B	109.5
H12A—C12—H12B	109.5	H24A—C24—H24B	109.5
C11—C12—H12C	109.5	C23—C24—H24C	109.5
H12A—C12—H12C	109.5	H24A—C24—H24C	109.5
H12B—C12—H12C	109.5	H24B—C24—H24C	109.5
			
C7—N1—C1—C2	−38.1 (8)	C19—N2—C13—C14	−35.7 (9)
C7—N1—C1—C6	147.9 (5)	C19—N2—C13—C18	146.8 (5)
C6—C1—C2—C3	−0.4 (7)	C18—C13—C14—C15	1.1 (8)
N1—C1—C2—C3	−174.3 (5)	N2—C13—C14—C15	−176.3 (5)
C1—C2—C3—C4	0.6 (7)	C13—C14—C15—C16	0.0 (7)
C11—O3—C4—C5	−179.0 (4)	C23—O6—C16—C15	2.1 (7)
C11—O3—C4—C3	2.0 (7)	C23—O6—C16—C17	−178.0 (4)
C2—C3—C4—C5	−0.7 (7)	C14—C15—C16—O6	178.9 (5)
C2—C3—C4—O3	178.2 (4)	C14—C15—C16—C17	−1.0 (7)
O3—C4—C5—C6	−178.5 (4)	O6—C16—C17—C18	−179.1 (4)
C3—C4—C5—C6	0.5 (7)	C15—C16—C17—C18	0.9 (7)
C4—C5—C6—C1	−0.3 (7)	C16—C17—C18—C13	0.2 (7)
C2—C1—C6—C5	0.3 (7)	C14—C13—C18—C17	−1.2 (8)
N1—C1—C6—C5	174.5 (5)	N2—C13—C18—C17	176.3 (5)
C1—N1—C7—O1	−0.7 (8)	C13—N2—C19—O4	−3.0 (9)
C1—N1—C7—C8	179.3 (5)	C13—N2—C19—C20	177.8 (5)
O1—C7—C8—C9	−82.6 (6)	O4—C19—C20—C21	−83.8 (6)
N1—C7—C8—C9	97.4 (5)	N2—C19—C20—C21	95.4 (5)
C7—C8—C9—O2	−10.0 (7)	C19—C20—C21—O5	−19.3 (8)
C7—C8—C9—C10	171.2 (5)	C19—C20—C21—C22	159.5 (5)
C4—O3—C11—C12	−174.0 (4)	C16—O6—C23—C24	−174.6 (4)

### Hydrogen-bond geometry (Å, º)

**Table:**

*D*—H···*A*	*D*—H	H···*A*	*D*···*A*	*D*—H···*A*
N1—H1*N*···O1^i^	0.92 (6)	1.98 (6)	2.878 (6)	165 (5)
N2—H2*N*···O4^ii^	0.89 (7)	1.98 (7)	2.856 (6)	168 (5)

Symmetry codes: (i) *x*, *y*−1, *z*; (ii) *x*, *y*+1, *z*.
